# Engineering the Crack Structure and Fracture Behavior in Monolayer MoS_2_ By Selective Creation of Point Defects

**DOI:** 10.1002/advs.202200700

**Published:** 2022-05-29

**Authors:** Gang Wang, Yun‐Peng Wang, Songge Li, Qishuo Yang, Daiyue Li, Sokrates T. Pantelides, Junhao Lin

**Affiliations:** ^1^ Department of Physics and Shenzhen Key Laboratory of Advanced Quantum Functional Materials and Devices Southern University of Science and Technology Shenzhen 518055 China; ^2^ School of Physics and Electronics Hunan Key Laboratory for Super‐Micro Structure and Ultrafast Process Central South University 932 South Lushan Road Changsha 410083 China; ^3^ Department of Physics and Astronomy and Department of Electrical and Computer Engineering Vanderbilt University Nashville TN 37235 USA

**Keywords:** atomic imaging, crack propagation, fracture behavior, monolayer MoS_2_, point defects

## Abstract

Monolayer transition‐metal dichalcogenides, e.g., MoS_2_, typically have high intrinsic strength and Young's modulus, but low fracture toughness. Under high stress, brittle fracture occurs followed by cleavage along a preferential lattice direction, leading to catastrophic failure. Defects have been reported to modulate the fracture behavior, but pertinent atomic mechanism still remains elusive. Here, sulfur (S) and MoS*_n_* point defects are selectively created in monolayer MoS_2_ using helium‐ and gallium‐ion‐beam lithography, both of which reduce the stiffness of the monolayer, but enhance its fracture toughness. By monitoring the atomic structure of the cracks before and after the loading fracture, distinct atomic structures of the cracks and fracture behaviors are found in the two types of defect‐containing monolayer MoS_2_. Combined with molecular dynamics simulations, the key role of individual S and MoS*_n_* point defects is identified in the fracture process and the origin of the enhanced fracture toughness is elucidated. It is a synergistic effect of defect‐induced deflection and bifurcation of cracks that enhance the energy release rate, and the formation of widen crack tip when fusing with point defects that prevents the crack propagation. The findings of this study provide insights into defect engineering and flexible device applications of monolayer MoS_2_.

## Graphical abstract

Sulfur and MoS*_n_* point defects are selectively created in various densities on suspended monolayer MoS_2_ using helium‐ and gallium‐ion‐beam lithography, subsequently followed by AFM nanoindentation test. Atomic scale imaging revels that different point defects lead to distinct fracture structures and mechanical characteristics. Combined with molecular dynamics simulations a defect‐induced toughening mechanism due to crack deflection and bifurcation is unveiled.

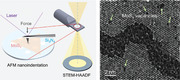

## 1 Introduction

Monolayer MoS_2_, a direct‐band‐gap semiconductor, has shown excellent application prospects in the fields of transistors,^[^ ^1^, ^2^, ^3^ ^]^ photodetectors,^[^ ^4^, ^5^ ^]^ and electroluminescent devices^[^ ^6^, ^7^ ^]^ and has become one of the most popular 2D materials in the post‐graphene era. Large‐scale synthesis of monolayer MoS_2_ at wafer size is now possible.^[^ ^8^, ^9^, ^10^ ^]^ Defect‐engineering investigations have found that purposely introducing 4.7% sulfur (S) vacancies in monolayer MoS_2_ can improve the carrier mobility and the on/off ratio of monolayer‐MoS_2_‐based transistors.^[^ ^2^ ^]^ High concentrations of S vacancies expose numerous coordinately unsaturated Mo atoms, which greatly enhance the electrocatalytic activity of MoS_2_ for the hydrogen‐evolution reaction.^[^ ^11^, ^12^ ^]^ Molecular dynamics (MD) simulations have also revealed that Mo‐terminated MoS_6_ vacancies are an ideal nanofiltration pore, which can effectively reject hydrated ions and allow water molecules to permeate at a high rate.^[^ ^13^ ^]^ More recently, the S vacancies created in a monolayer MoS_2_ has been demonstrated to have high optical activity and can stably produce photons in the visible spectral range as single defect photon‐emitters, which originates from the excitonic transition between the defect orbits.^[^ ^14^, ^15^ ^]^ The existence of atomic point defects destroys the local structural symmetry of monolayer MoS_2_ and produces excellent piezoelectric effect.^[^ ^16^ ^]^ The external strain can modulate the band structure of monolayer MoS_2_, and thus adjust the photoluminescence to shift red or blue.^[^ ^17^, ^18^ ^]^ Therefore, defect engineering and strain engineering have brought new development opportunities for promising applications of monolayer MoS_2_.

In recent years, owing to the progress of micro‐nano manufacturing technology, irradiation by gallium‐ion (Ga^+^) and helium‐ion (He^+^) beams has been successfully used to create defects in 2D materials.^[^ ^14^, ^19^, ^20^, ^21^, ^22^, ^23^, ^24^ ^]^ Compared with H_2_ annealing,^[^ ^2^, ^25^ ^]^ plasma treatment,^[^ ^26^ ^]^ and other methods, ion‐beam lithography can achieve a continuous adjustment of defect size and concentration within a certain range, and, more importantly, can achieve selective etching at the atomic level. Studies have confirmed that irradiation of monolayer MoS_2_ by Ga^+^ mainly produces MoS*_n_* defects,^[^ ^22^, ^23^, ^24^, ^27^ ^]^ while irradiation by He^+^ with a smaller atomic radius and mass predominantly generates S vacancies.^[^ ^12^, ^21^ ^]^ However, controllable introduction of defects into the periodic lattice would affect the macroscopic mechanical properties, which are the premise for realistic applications. To further advance defect engineering of 2D materials, it is urgent to establish the correlation between the defect structures and the mechanical behaviors of defective 2D materials, preferentially under load and stress that mimics realistic working environments.

Previous studies have widely demonstrated that pristine MoS_2_ has excellent strength and Young's modulus comparable to steel,^[^ ^28^, ^29^, ^30^ ^]^ but its fracture toughness is insufficient.^[^ ^31^, ^32^ ^]^ Under a shear force, Mo—S bonds are broken and cleavage occurs along the zigzag or armchair direction, forming long and straight lattice edges.^[^ ^33^ ^]^ For defect‐containing MoS_2_ monolayers, however, limited studies have been reported on how defects affect the mechanical properties at the atomic scale. A recent study shows that MoS_2_ treated with Ga^+^ and argon ions (Ar^+^) features shorter crack propagation distances and exhibits considerable toughness enhancement, but there exists no detailed atomic‐scale evidence on the origin of defect‐induced enhancement of fracture toughness.^[^ ^27^ ^]^ Moreover, both Ar^+^ and Ga^+^ generate the same defect type in MoS_2_ due to similar atomic radii, which makes it hard to correlate the fracture behavior with different types of defects. An alternative approach is to use electron bombardment to generate S defects at the crack tip and provide energy for continuous propagation of the crack. A dynamical atomic view on the effect of S defects during crack propagation is provided by simultaneously atomic (scanning) transmission electron microscopy ((S)TEM) imaging.^[^ ^31^, ^33^, ^34^ ^]^ However, the driving force that initiates fracture is local beam‐induced stress which cannot be equivalent to the real load and tension, and the continuous knock‐on damage from electron irradiation to the imaged lattice may also affect the dynamical fracture process, i.e., different mechanism may dominate in realistic catastrophic failure by high load. Therefore, under external loading, the detailed atomic scenario of fracture behavior affected by different types of defects in monolayer MoS_2_ is still missing.

In the present work, He^+^ and Ga^+^ lithography were used to selectively generate dominant S and MoS*_n_* vacancies in suspended monolayer MoS_2_, respectively. We systematically studied the variation of concentration, size, and average spacing of these defects as a function of the ion‐beam irradiation dose, which provides guidance and selection basis for the subsequent mechanical test. We then used AFM nanoindentation experiments to reveal the mechanical properties of the MoS_2_ monolayers that have been engineered with specific types of defects. Through a combination of high‐resolution STEM observations before and after the mechanical loading and MD simulations, we establish the atomic correlation between the specific type of defect structure and the fracture behaviors. More importantly, we also derive insights into the modulation of crack propagation behavior induced by different type of defects. Moreover, based on the stress analysis at the crack tip structure, we further provide a fracture‐toughening scheme in terms of inhibiting crack propagation by defect‐induced crack blunting.

## 2 Results and Discussion

**Figure 1:**
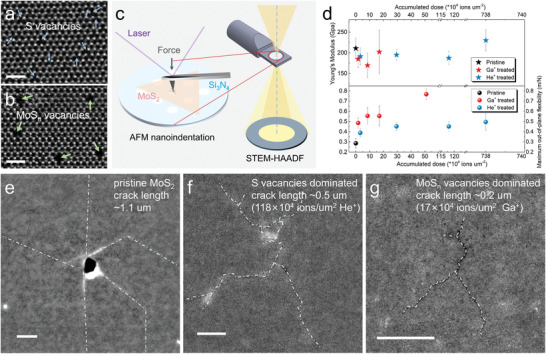
Mechanical properties of pristine/defective monolayer MoS_2_ with selective defects and STEM characterization of the fracture traces after indentation test. HAADF‐STEM images of monolayer MoS_2_ with a) dominated S_2_ vacancies created by 30 kV He^+^ irradiation, and b) dominated MoS*_n_* vacancies created by 30 kV Ga^+^ irradiation. The scale bars in (a) and (b) are 1 nm. c) Schematic of nanoindentation on suspended MoS_2_ flake and subsequent transfer to STEM characterizations. d) The statistics results of Young's modulus and maximum out‐of‐plane flexibility of monolayer MoS_2_ in pristine state and treated with different doses of Ga^+^ and He^+^. Low magnification STEM images of the fracture traces after mechanical test of e) pristine monolayer MoS_2_, f) He^+^‐irradiated monolayer MoS_2_, and g) Ga^+^‐irradiated monolayer MoS_2_. The scale bars in (e)–(g) are 100 nm.

Monolayer MoS_2_ flakes grown by chemical vapor deposition (CVD) were transferred to a perforated silicon nitride (Si_3_N_4_) TEM window with a thickness of 100 nm, for both the AFM nanoindentation tests and high‐resolution STEM experiments. Different doses of He^+^ and Ga^+^ were used to irradiate the suspended monolayer MoS_2_ to create defects (see schematic illustration in Figure S1a,b, Supporting Information). Aberration‐corrected low‐voltage high‐angle angular‐dark‐field STEM (HAADF‐STEM) was used to systematically reveal the dose‐dependent defect structure in suspended monolayer MoS_2_ after He^+^ and Ga^+^ irradiation. Atomic resolution, high‐magnification STEM images show that moderate He^+^ irradiation (29.5 × 10^4^ ions µm^−2^) mainly produces S and S_2_ vacancies, while moderate Ga^+^ irradiation (17 × 10^4^ ions µm^−2^) mostly yields MoS*_n_* vacancies (*n* = 3–6), as shown in **Figure** **1**a,b and Figure S1c–e (Supporting Information). These particular defects occur because the collision cross‐section of Ga^+^ and He^+^ at Mo and S sites are quite different owing to the atomic radii of incident ions,^[^ ^35^ ^]^ as illustrated in Figure S1a,b (Supporting Information).

In addition, increasing the dose of He^+^ irradiation leads to higher concentrations of S and S_2_ vacancies, and, when the dose reaches 118 × 10^4^ ions µm^−2^, sparse MoS*_n_* vacancies also appear and become denser as the ion dose is increased further. On the contrary, increasing the dose of Ga^+^ irradiation mainly increases the concentration of MoS*_n_* defects and reduces their spacing. When the dose of Ga^+^ irradiation is increased to 51 × 10^4^ ions µm^−2^, nanoscale holes are also generated. Figure S2 (Supporting Information) shows a series of atomic‐resolution STEM images of defective MoS_2_ monolayers treated by varying the ion doses and the statistical evolution of the MoS*_n_* and S/S_2_ vacancy concentrations as a function of the total ion irradiation dose. The corresponding low‐magnification STEM images are shown in Figures S3 and S4 (Supporting Information). Therefore, by controlling the ion dose, pervasive S and MoS*_n_* vacancies can be selectively created, providing the premise for investigating their individual contributions to the mechanical behavior.

In order to reveal the effects of different types and concentrations of point defects on the mechanical properties of monolayer MoS_2_, a vertical load (see schematic illustration in Figure 1c) was applied to suspended pristine/defective MoS_2_ membranes at a fixed loading velocity through an AFM probe until fracture occurred. Force (*F*) versus Indentation (*δ*) curves were obtained in real time, as partially shown in Figure S5b (Supporting Information). The AFM probe is placed at the center of the circular hole in a Si_3_N_4_ window. The stress applied to the membrane suspended on the hole is isotropic. In addition, the radius of the AFM tip is much smaller than that of the hole, which is consistent with the previously reported 2D material nanoindentation experimental model.^[^ ^30^, ^36^, ^37^ ^]^ Therefore, the measured *F*–*δ* curve can be well fitted by the following cubic polynomial

(1) $$F=\sigma_{0}^{2D}\pi \delta +E^{2D}\frac{q^{3}\delta^{3}}{r^{2}}$$

Here, $\sigma_{0}^{2D}$ represents prestrain, *E* ^2D^ is the 2D elastic modulus, *r* denotes the hole radius of Si_3_N_4_, and *q* is a constant determined by the Poisson's ratio *ν* of the material, given by *q* = (1.05 − 0.15*ν* − 0.16*ν* ^2^)^−1^. Based on Equation (1) and the known membrane thickness *t* (0.65 nm for monolayer MoS_2_), the corresponding Young's modulus can be calculated by

(2) $$E_{Y}=\frac{E_{2D}}{t}$$

The fracture of a 2D membrane occurs at the discontinuity of the *F*–*δ* curve (marked in Figure S5b, Supporting Information). When fracture happens and a crack is generated, the contact zone between the AFM tip apex and the 2D membrane bear the maximum load and strain, and the pertinent maximum out‐of‐plane flexibility ($\lambda_{\max}^{z}$) and fracture strength ($\sigma_{\max}^{2D}$) can be calculated based on

(3) $$\lambda_{\max}^{z}=\frac{\delta }{F},$$

(4) $$\sigma_{\max}^{2D}={\left(\frac{F_{\max}E^{2D}}{4\pi r_{\mathrm{tip}}}\right)}^{\frac{1}{2}}$$

Equation (4) has been widely used to evaluate the fracture strength of 2D materials.^[^ ^27^, ^36^, ^37^ ^]^

The *E*_Y_ of pristine monolayer MoS_2_ measured in our experiment is 211  ±  25 GPa and the breaking strength is 15  ±  2 N m^−1^, which is consistent with previous reports.^[^ ^30^, ^38^ ^]^ The same method was used to measure defective monolayer MoS_2_ membranes engineered with specific S and MoS*_n_* vacancy defects in different concentrations. The *E*_Y_, $\lambda_{\max}^{z}$ and breaking strength of pristine, S‐vacancy‐enriched (He^+^‐irradiated) and MoS*_n_*_‐_vacancy‐enriched (Ga^+^‐irradiated) MoS_2_ monolayer are depicted in Figure 1d and Figure S5c (Supporting Information), respectively. The $\lambda_{\max}^{z}$ of defective monolayer MoS_2_ membranes increases while the breaking strength decreases monotonically with increasing defect density, in which the breaking strength treated by Ga^+^ decreases more drastically. Compared with pristine MoS_2_ membrane, the *E*_Y_ of defect‐enriched MoS_2_ membranes also show downward trends with increasing defect concentration, representing the decrease of material stiffness and the improvement of internal flexibility. The abnormal rise of the fitted *E*_Y_ (greater than 300 GPa) of MoS_2_ membranes irradiated with very‐high‐dose ions, especially high‐dose Ga^+^ (51 × 10^4^ ions µm^−2^), is attributed to the deviation between the measured force curve and the fitting model (no longer a continuous film). This result is evidenced by the discrete nanoscale holes that are generated at such high Ga^+^ irradiation doses (Figure S4, Supporting Information), thereby the fitted cubic term is no longer credible, which is also reflected in the surge of errors (Figure 1d).

After AFM indentation‐force measurements, the fractured specimens were immediately reloaded into an aberration‐corrected TEM for atomic‐resolution crack structure analysis. The low‐magnification STEM images in Figure 1e show that a pristine MoS_2_ membrane after fracture has long (>1µm), straight, and sharp cracks, which geometrically conform to the characteristics of a Griffith brittle crack.^[^ ^39^ ^]^ In sharp contrast, rough crack edges, frequent crack deflection and bifurcation are observed in the moderate He^+^‐ and Ga^+^‐irradiated MoS_2_ membranes as shown in Figure 1f,g, while the propagation lengths of cracks are significantly reduced to about 0.5 and 0.2 µm, respectively. These crack propagation behaviors, which are quite different from those of pristine MoS_2_ membranes, are generally considered to be the characteristics of ductile fracture.^[^ ^39^ ^]^

**Figure 2:**
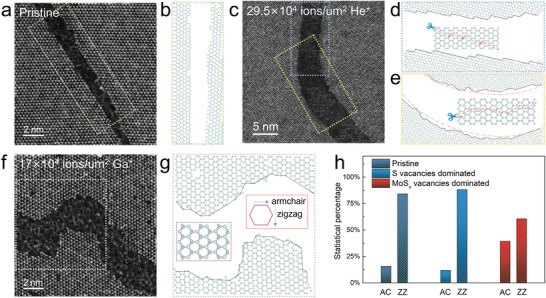
Atomic structure of cracks produced by indentation fracture measurement. a–e) The atomic resolution HAADF‐STEM images show the atomic structure of the crack in pristine (a) and moderate He^+^ (29.5 × 10^4^ ions/µm^2^) irradiated monolayer MoS_2_ (c). The corresponding atom‐by‐atom models of the a) green, c) blue and yellow highlighted regions are shown in (b), (d), and (e), respectively. The insets in (d) and (e) show a schematic of the ripping directions of Mo—S bonds during the occurrence of fracture at the corresponding regions. f) The HAADF‐STEM image of the crack in the monolayer MoS_2_ irradiated by high‐dose Ga^+^ (17 × 10^4^ ions µm^−2^). g) The corresponding atomic structure model of the white dashed box area in (f). The inset in (g) indicate the armchair direction and zigzag direction of MoS_2_ lattice. h) Statistics of crack propagation direction along armchair and zigzag directions.

Atomic‐resolution HAADF‐STEM imaging was used to reveal the atomic structure of the crack path in order to better understand how the defects modulate the propagation of cracks and alter the fracture mechanism of brittle materials. Fractured pristine MoS_2_ forms atomically sharp and clean cleavage edges along certain zigzag directions, due to the unzipping of Mo‐S bonds in the same row, as shown in **Figure** **2**a,b. An arc‐shaped crack appears in MoS_2_ membrane irradiated by He^+^, while a higher‐magnification atomic‐scale image shows step‐like edge terminations (Figure 2c). It can be deduced that the ordered step‐like edges at the straight region (Figure 2d, highlighted by dashed blue rectangle in Figure 2c) are formed by continuous deflection along the two zigzag directions while the crack is ripping Mo‐S bonds at the two sides. Nevertheless, the lower curved region (Figure 2e, highlighted by dashed yellow rectangle in Figure 2c) shows step‐like edges that are more disordered. We deduced that this feature is due to the crack being deflected along both the armchair and zigzag lattice direction as illustrated in the inset of Figure 2e, thus forming an arc‐shaped crack with large curvature.

MoS_2_ membranes irradiated by Ga^+^ frequently show more dramatic and disordered crack deflection after fracture, including even a sharp 90˚ deflection. The atomic‐scale mapping of the crack edge reveals that, these features are caused by propagation along the armchair lattice direction (Figure 2f,g) at a much higher frequency than the He^+^‐irradiated cases. The statistical results of the crack‐propagation direction show that nearly 85% of the cracks in pristine MoS_2_ propagate along the zigzag direction and that the propagation angle (*θ*) shows a six‐fold symmetry (Figure 2h and Figure S6, Supporting Information), an isotropic feature, when releasing the stress. The proportion of cracks propagating along the zigzag direction in He^+^‐irradiated MoS_2_ membrane is slightly higher than that of the pristine one, but the deflection frequency of the crack propagation is much higher. It follows that the existence of S vacancies damages the local isotropic bonding symmetry of MoS_2_, which guides the crack to rip the weaker Mo—S bonding at the defect sites, resulting in anisotropic propagation directions, but still follows the zigzag lattice (the minimum energy release direction). In sharp contrast, the propagation proportion of cracks in Ga^+^‐irradiated MoS_2_ along the armchair direction increases to about 40%, which suggest that the local lattice geometries and Mo‐S bonding symmetry are further destroyed, and the minimum energy release direction is not completely along the zigzag lattice.

**Figure 3:**
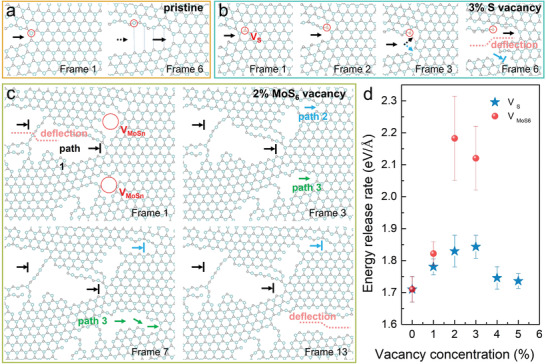
MD simulation on the fracture behaviors and the corresponding energy release rate in pristine and defective monolayer MoS_2_. Time series snapshots of the detailed propagation of the crack tip extracted in a) pristine MoS_2_, showing a cleavage fracture along the zigzag direction; b) fractured monolayer MoS_2_ with 3% S vacancy depicts the dynamical formation of a step‐like edge. The red circle highlights a single S vacancy alongside the crack, which deflect the crack to form a step; c) fractured monolayer MoS_2_ with 2% MoS_6_ vacancy shows frequent deflections and changes of the crack path. The red circles emphasize the position of MoS*_n_* vacancies, and two new propagation paths appear next to them, which bifurcates the original crack. d) The calculated energy release rate as a function of S and MoS_6_ vacancy concentration.

To correlate the atomic‐scale experimental observations with the mechanism, large‐scale atomistic MD simulations are performed. As a trial test, MD simulations successfully reproduce the atomically sharp and clean cleavage of pristine MoS_2_ along the zigzag lattice direction. Edge energy calculations suggest the zigzag lattice direction is the minimum energy release direction. We then further investigate the fracture behavior by creating different types of defects in the model, which also yield out rough, step‐like crack edges, as observed in S‐vacancy enriched MoS_2_, and high‐frequency crack bifurcation in MoS_6_‐vacancy‐enriched MoS_2_. Time series of snapshots showing the detailed chemical bond‐breaking process during the crack propagation in both pristine and different defective MoS_2_ monolayers are presented in **Figure** **3**a–c, with final fractured structures shown in Figure S7 (Supporting Information). In pristine MoS_2_, the Mo atom marked by a red circle and the S atom below it are gradually separated under tensile stress until the Mo‐S bond breaks. As the crack propagation, the distance between the Mo—S gradually increases, and more Mo‐S bonds ahead of them gradually break, finally forming a sharp zigzag edge along the black arrow, which confirms that the zigzag lattice is the minimum energy‐release direction.

Under the same tensile stress, however, the crack propagation depicted in Figure 3b deviates from the original path, forming a step‐like edge. Compared with pristine monolayer MoS_2_, the absence of S atoms reduces the number of Mo—S covalent bonds and weakens their angular stiffness, allowing greater local deformation. The result is anisotropic lattice distortions near the crack tip (see the atomic structure and bond length distribution in Figure S8, Supporting Information). Therefore, when the crack extends to the S vacancy marked by the red circle, a single Mo—S bond is torn first under stress and the crack then deflects to the right front along the black dotted arrow, resulting in a step‐like deflection. In addition, the asymmetric stress distribution at the crack tip leads to the break of some nearby Mo—S bonds after excessive deformation and the bifurcated crack propagates for a distance in the direction shown by the blue dotted arrow and terminates, forming a section of a microcrack.

On the other hand, when MoS_6_ vacancies that are larger in size are introduced, the deformability of the structure is further enhanced, which strongly modulates the local strain distribution. As the crack propagates, the breaking of the local chemical bonds changes the minimum energy release direction, jointly giving rise to the deflection, jump and bifurcation of the crack path, and finally generate the network microcrack structure as shown in Figure 3c. Figure 3d shows the variation of energy release rate (*G*_C_) with respect to the defect concentration of S vacancies and MoS_6_ vacancies. According to the classic Griffith theory, the critical strain energy release rate of fracture determines the fracture toughness of a mechanical sample. It can be clearly seen that as the concentration of S vacancies increases, the energy release rate of monolayer MoS_2_ increases from 1.7 eV Å^−1^ (pristine) to 1.85 eV Å^−1^ (3% S vacancies), and then slowly decreases. The existence of MoS_6_ vacancies has a greater impact on the energy release rate of monolayer MoS_2_, which reaches as high as 2.2 eV Å^−1^ when the MoS_6_ vacancy concentration is up to 2%. Therefore, point defects induced large number of microcracks, which rapidly dissipate strain energy through crack deflection and bifurcation, giving rise to much shorter propagation crack length in defective MoS_2_. Table S1 (Supporting Information) summarizes the average crack lengths after fracture and estimated mechanical characteristics of MoS_2_ membranes treated with different doses of He^+^ and Ga^+^. An obvious trend is that the crack length decreases with the increase of vacancy concentration (both S and MoS*_n_* types). This indicates that the increased defect density localizes the crack propagation due to the increased energy dissipation per unit length, further supporting our proposed mechanism. Note that the calculated critical stress intensity factor (*K*_IC_), which is directly associated to the bonding strength at the crack tip, decreases with the increase of vacancy concentration (Figure S9, Supporting Information). This suggests that energy release rate is compensated in the pristine case, but still in a negligible level due to the limited number of cracks created after the loading fracture.

**Figure 4:**
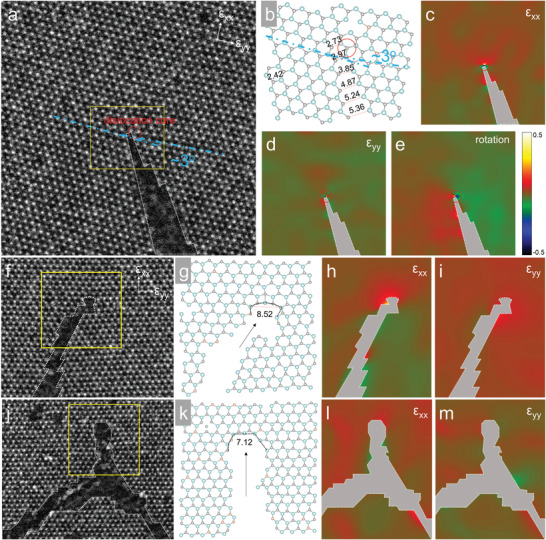
Atomic structure and local stress analysis of different crack tip structures. a) High‐magnification HAADF‐STEM image of an atomically sharp crack, and b) the atomic structure model at the crack tip, corresponding to the yellow box area in (a). The Mo—S bond lengths along the crack are labeled on the model. GPA strain analysis of the STEM image and corresponding strain components c) *ε*_xx_, d) *ε*_yy_, and e) rotation, showing localized strain around the dislocation core highlighted in red circle. f–k) HAADF‐STEM images of widened crack tip observed in defective monolayer MoS_2_ with dominating f) S vacancies and j) MoS*_n_* vacancies defects. The atomic mapping models of the yellow box areas in (f) and (j) are shown in (g) and (k), respectively. GPA strain analysis of the axial strain components h,l) *ε*_xx_ and i,m) *ε*_yy_ of the widen crack front shown in (f) and (j), respectively, showing that the strain has been released by the blunt crack tip structure.

Another experimental observation is that the fracture length in MoS*_n_*‐vacancy‐dominated defective MoS_2_ monolayers is nearly 60% shorter than the S‐vacancy dominated monolayers, which may not be solely interpreted by the enhanced energy release rate (2.2 vs 1.85 eV Å^−1^, only 20% higher). In a fracture event, the maximum stress and strain field near the crack tip determines the continuous propagation or termination of the crack. The geometric phase analysis (GPA) of atomic‐resolution HAADF images is used to observe the residual strain distribution at the crack tips with different atomic geometry. **Figure** **4**a,b show an atomically sharp crack tip formed by the gradual stretching and breakage of Mo—S bonds along the zigzag direction. The distribution of the axial strains *ε*_xx_, *ε*_yy_ and lattice rotation extracted by GPA are shown in Figure 4c–e, demonstrating the centralization of residual stress near the crack tip. A highly strained dislocation core is formed at the tip which is highlighted by red circles, weakening the surrounding Mo—S bonds that facilitate the continuous propagation of cracks. In defective MoS_2_ membranes, however, we observed frequently widened crack tips where the front of the crack has a length several times wider than the Mo—S bond length. GPA analysis of these widen crack tips region shows a dispersed axial strain distribution compared with the atomically sharp crack tip (Figure 4f–m). This result suggests that the accumulated stress is released at the front of the crack whenever such structure is formed, thereby preventing the further propagation of the brittle crack.

**Figure 5:**
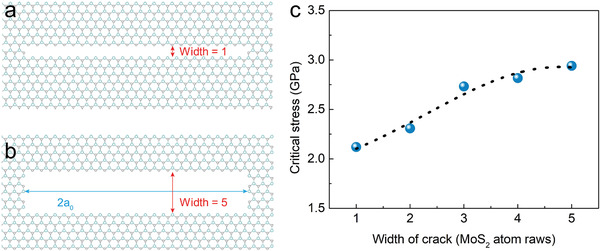
MD simulation of the blunt crack structure and the corresponding critical stress. a,b) Atomic structure of MoS_2_ monolayer slabs with 1 row and 5 rows of MoS_2_ zigzag chains removed from the center crack. The removed rows in the pre‐crack model mimics a widen crack tip structure. c) The calculated critical fracture stress as a function of the crack tip width, showing an enhanced critical stress as the width of the crack tip increase, suggesting a blunt widen crack tip is more robust against crack propagation.

The above mechanism is also verified by MD simulations, which directly evaluate the effect of crack blunting on the critical fracture stress of 2D materials. As shown in **Figure** **5**a,b, one to five rows of MoS_2_ atomic chains along the zigzag direction are removed to create pre‐crack tips with different widths, while remaining the same crack length of 2*a*_0_ = 8 nm. Figure 5c depicts the trend of monotonic increase of critical fracture stress with crack width. When the width of the crack tip expands from one row to five rows of MoS_2_ chains, the critical stress increases from 2.12 to 2.94 GPa. Thus, the widened crack tip structure can effectively increase the critical fracture stress, improve the fracture toughness, so as to prevent the brittle crack propagation. Moreover, these widened crack tips are formed by the fusion of the propagating crack when they encounter vacancy defects followed by subsequent atomic reconstruction, and are much easier to form when fusing with MoS*_n_* vacancies, which have larger size than single S vacancies as evidenced in Figure 4j,k. This feature accounts for the much shorter crack length observed in the MoS*_n_*‐dominated defective MoS_2_ monolayer, which has higher probability to stop the crack by blunting it with fusion of MoS*_n_* vacancies.

## 3 Conclusion

We demonstrated that the irradiation by He^+^ and Ga^+^ can selectively create high‐density S and MoS*_n_* vacancy point defects in suspended MoS_2_ monolayers. By synergistically combining the AFM nanoindentation technique with STEM atomic‐structure characterization, the effects of different types of point defects on the mechanical properties and fracture behavior of monolayer MoS_2_ were revealed. By analyzing the atomic structure of crack edges induced by different defects, we found that the defect‐induced lattice symmetry breakage promotes the crack deflection or bifurcation, which improves the fracture toughness by enhancing the energy release rate in the fracture process. MD simulations and local strain distribution analysis at the crack tip region show that fusion with defects blunts the crack front and is conducive to prevent brittle crack propagation. Our results provide an atomic understanding of fracture mechanics of 2D materials, and offer an idea for shortening the brittle crack. Although previous works have demonstrated the application prospects of 2D defect engineering in some cutting‐edge fields, such as single photon emitters,^[^ ^14^, ^15^ ^]^ ultradense storage media,^[^ ^40^, ^41^, ^42^ ^]^ flexible electronics,^[^ ^16^, ^43^ ^]^ defect associated qubits,^[^ ^44^, ^45^ ^]^ it is undeniable that the 2D defect engineering toward realistic application is still in its infancy. Our work will provide insights for the subsequent optimization and scale application of these functions, such as maximizing the density of defects without compromising the mechanical stability, and rationally selecting special flexible substrates to match the requirements of toughness and strength. For these promising functions to be on the horizon, apart from the study of local defect structures and associated electronic states, the research community should not ignore the relatively blank research on the mechanical stability, which is also the basis of 2D defective materials for large‐scale practical application.

## 4 Experimental Section

### CVD Synthesis and Transfer of Monolayer MoS_2_ Crystal

The dual‐temperature zone tube furnace was used to synthesize monolayer MoS_2_ flakes. The detailed growth parameters can be found in the ref. ^[^ ^46^ ^]^. MoS_2_ flakes grown on the SiO_2_ substrate were transferred to a perforated Si_3_N_4_ TEM window using a PMMA‐assisted method. First, a PMMA film with a thickness of about 200 nm was spin‐coated on the SiO_2_ surface at 4000 rpm, and it was floated in 2 m NaOH solution at 80 °C afterward. After a few hours, the PMMA/MoS_2_ film peeled off from the substrate, and then the film was carefully transferred to deionized water for rinsing several times. The perforated Si_3_N_4_ TEM window was used to fish up the PMMA film, which was then dried and immersed in acetone overnight. Finally, the Si_3_N_4_ TEM window was annealed in a high vacuum annealing furnace at 230 °C for 6 h.

### Ga^+^ Beam and He^+^ Beam Irradiate Suspended Monolayer MoS_2_

Suspended monolayer MoS_2_ flakes were irradiated with Ga^+^ and He^+^ accelerated by 30 kV voltage using a helium ion microscope (HIM, Carl Zeiss, Orion nanofab). For Ga^+^ irradiation, the beam current was set to ≈3 pA, and the dwell time and repeats time were fixed to 0.1u s and one time, while pixel spacing X and Y were adjusted to realize the continuous change of irradiation doses in raster mode. Patterns with an area of 3 µm × 3 µm were selectively drawn on Si_3_N_4_ holes suspended with monolayer MoS_2_, and then irradiated with different doses of Ga^+^ to generate different atomic defects. The similar parameters were used for He^+^ treatment, except that the current of He^+^ was increased to ≈300 pA. Note that the total irradiation dose per unit area determines the final defect type and density, which is associated with irradiation current and time. That is to say, one can achieve the same effect on defect creation by reducing current and increasing irradiation time.

### AFM Force Curve Measurement

Indentation fracture measurement was performed in an Oxford Cypher S AFM controlled with Asylum Research software. SD‐R150‐NCL and AC200TS AFM probes were used for nanoindentation measurement, and the spring constant of the AFM cantilever was calibrated by thermal noisy method prior to each experiment. Afterward, the tapping mode was used to image the morphology of the flakes and confirm the target position to load the AFM tip. Last, the tip was controlled to press into the suspended membranes at a loading velocity of 200 nm s^−1^ in contact force mode until fracture occurred.

### STEM Characterization

HAADF‐STEM images were acquired on an FEI Titan Themis G2 double‐ aberration‐corrected TEM operating at 60 kV. The collection angle of HAADF detector was around 52–200 mrad, and the convergence semi angle was 25 mrad. The beam current was set about 30 pA for the annular dark‐field imaging to reduce the electron beam induced damage.

### MD Simulations

MD simulations were conducted using the Large‐scale Atomic/Molecular Massively Parallel Simulator (LAMMPS) package. The classical force field developed by Liang et al.^[^ ^47^, ^48^, ^49^ ^]^ with the cutoff distances modified as suggested by Wang et al.^[^ ^33^ ^]^ was employed for describing the inter‐atomic interactions. A rectangular simulation region was filled by the MoS_2_ monolayer with the *x*‐direction aligned to the zigzag direction of monolayer MoS_2_. S vacancies and MoS_6_ vacancies were created by randomly deleting them from the system. For each defect concentration, five samples were generated in order to count for the randomness in the defect positions. A diamond‐shape hole was digged at the center of the simulation region to initialize the fracture. MD simulations was run using the NPT ensemble with the temperature set to 1 K and the pressure along the *x*‐direction set to zero. The atomic structure and the size of the simulation cell was fully optimized using a NPT simulation; this state is referred to as the initial state. Starting from the initial state, a strain was applied along the *y*‐direction with the increasing rate of the strain set to 7.5 × 10^−5^ ps^−1^. When the strain reaches ≈10%, the fracture propagates through the whole sample and the sample breaks completely; this state is referred to as the final state. The MD trajectory as well as the stress along the *y*‐direction were recorded for further analysis. The energy release rate (*J*) was calculated from the total energy of the initial state (*E*_i_) and the total energy of the final state (*E*_f_) as, *J* = (*E*_f_ – *E*_i_)/*L*_x_, where *L*_x_ is the length of simulation cell along the *x*‐direction. MD simulations were conducted with a series of different *L*_x_. The value was extracted from a linear fitting of the total energy difference (*E*_f_ – *E*_i_) versus L_x_.

### Statistical Analysis

Atomic‐resolution HAADF‐STEM images collected in different regions were used to calculate the defect density of monolayer MoS_2_ treated with different doses of ions. CalAtom software^[^ ^50^ ^]^ was used to analyze the atomic intensities of these HAADF images, and the lattice atomic sites with intensity lower than the background noise of HAADF detector are considered as vacancies. At each ion dose, more than five HAADF images were used for counting, with an area of more than 100 nm^2^_._ Note that the S vacancies refer to those isolated and dispersed S and S_2_ vacancies, excluding the S atoms originally bonded to the lost Mo atoms, which are assigned to MoSn vacancies together. The statistics of crack propagation direction along armchair and zigzag directions were also based on more than three atomic‐resolution HAADF images, and were manually counted according to the results of Z‐contrast judgment.

## Conflict of Interest

The authors declare no conflict of interest.

## Supporting information

Supporting Information

## Data Availability

The data that support the findings of this study are available from the corresponding author upon reasonable request.
