# Supplementary material for: Engineering the Crack Structure and Fracture Behavior in Monolayer MoS2 By Selective Creation of Point Defects
Source: Adv Sci (Weinh). 2022 May 29;9(22):2200700. doi: 10.1002/advs.202200700 (PMC9353506; doi:10.1002/advs.202200700)
Supplement: Supplementary file 1 — Supporting Information [file ADVS-9-2200700-s001.pdf]

ADVANCED SCIENCE
Open Access

Supporting Information

for *Adv. Sci.*, DOI 10.1002/advs.202200700

Engineering the Crack Structure and Fracture Behavior in Monolayer MoS_2 By Selective Creation of Point Defects

*Gang Wang, Yun-Peng Wang, Songge Li, Qishuo Yang, Daiyue Li, Sokrates T. Pantelides and Junhao Lin**

Supporting information

Engineering the crack structure and fracture behavior in monolayer MoS_2 by selective creation of point defects

Gang Wang[1,†], Yun-Peng Wang[2,†], Songge Li[1], Qishuo Yang[1], Daiyue Li[1], Sokrates T. Pantelides,[3] Junhao Lin[1,*]

[1]Department of Physics and Shenzhen Key Laboratory of Advanced Quantum Functional Materials and Devices, Southern University of Science and Technology, Shenzhen 518055, China

[2]School of Physics and Electronics, Hunan Key Laboratory for Super-Micro Structure and Ultrafast Process, Central South University, 932 South Lushan Road, Changsha 410083, People's Republic of China

[3]Department of Physics and Astronomy and Department of Electrical and Computer Engineering, Vanderbilt University, Nashville, TN 37235, USA

†These authors contributed equally to this work.

[*]Corresponding author: linjh@sustech.edu.cn

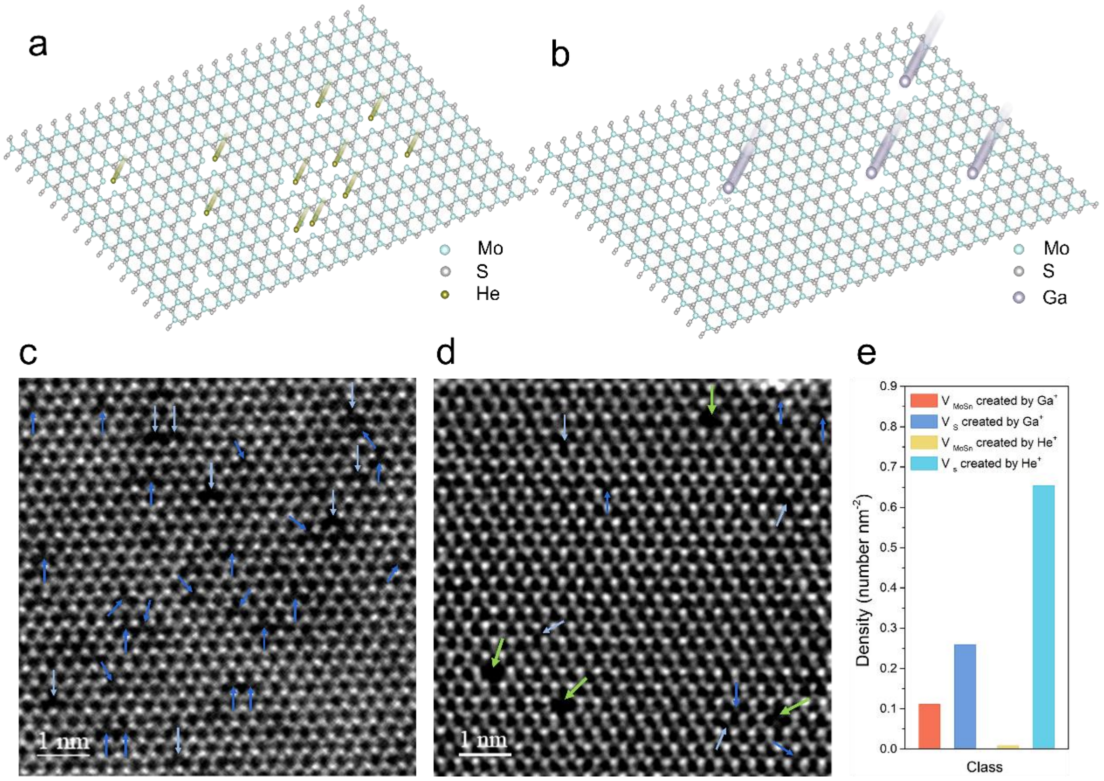

Figure S1. Schematic and experimental images of He^+ and Ga^+ irradiated suspended monolayer MoS_2, as well as defects statistics. Schematic shows that the MoS_2 monolayer is irradiated by (a) dispersed He^+ ions to generate numerous S/S_2 column vacancies, and (b) dispersed Ga^+ to generate numerous MoS_n vacancies. Atomic-resolution STEM images of representative monolayer MoS_2 after irradiated by (c) He^+ and (d) Ga^+. The light blue, blue and green arrows indicate single S_2 column vacancies, single S vacancies and MoS_n vacancies, respectively. (e) The average density of S/S_2 and MoS_n vacancies are based on the statistical results of three atomic-resolution STEM images with area over 50 nm^2.

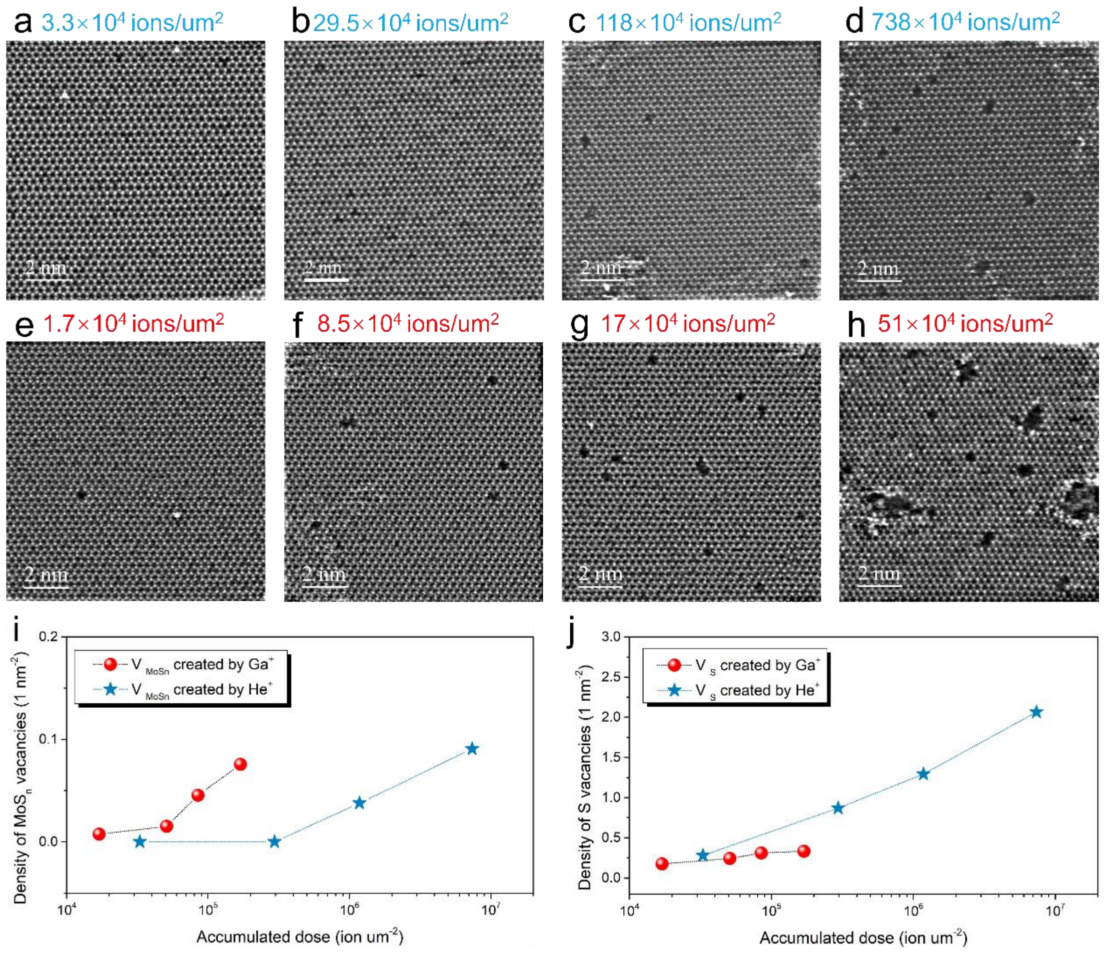

Figure S2. High-magnification HAADF-STEM images of suspended monolayer MoS_2 irradiated by 30 kV He^+ and Ga^+ with different doses of ion, as well as statistical results of defects created by various doses of ion. HAADF-STEM images of monolayer MoS_2 exposed to He^+ irradiation with doses of (a) 3.3×10^4 ions μm^{-2}, (b) 29.5×10^4 ions μm^{-2}, (c) 118×10^4 ions μm^{-2}, (d) 738×10^4 ions μm^{-2} and Ga^+ irradiation with doses of (e) 1.7×10^4 ions μm^{-2}, (f) 8.5×10^4 ions μm^{-2}, (g) 17×10^4 ions μm^{-2}, (h) 51×10^4 ions μm^{-2}. Statistical density of (i) MoS_n vacancies and (j) S/S_2 vacancies created by He^+ and Ga^+ with various doses of ion.

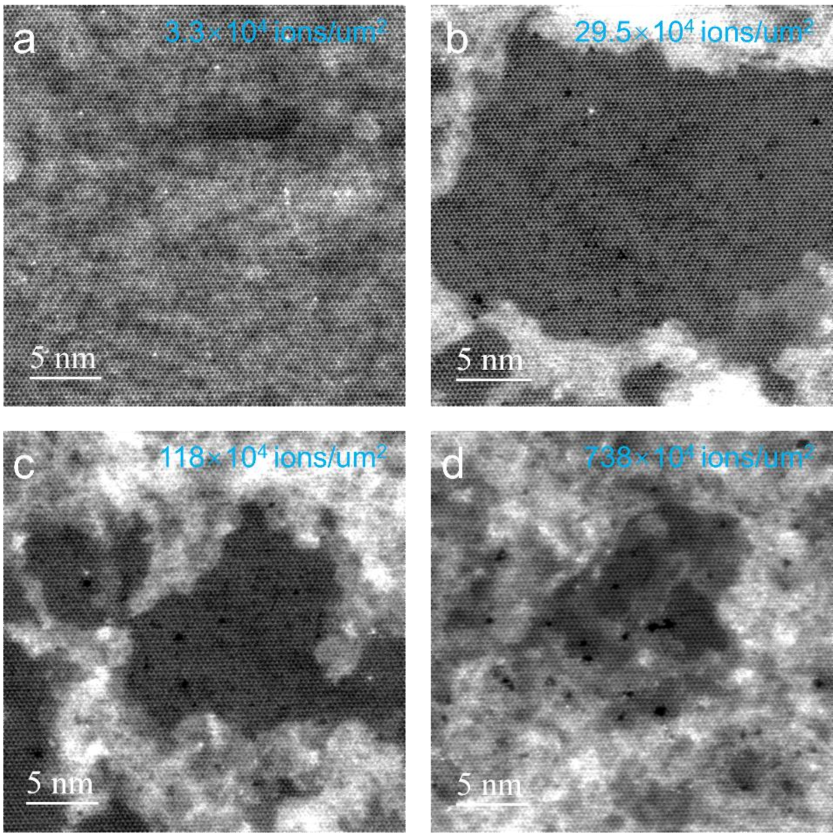

Figure S3. Low magnification HAADF-STEM images of suspended monolayer MoS_2 irradiated by 30 kV He^+ with different doses of ion.

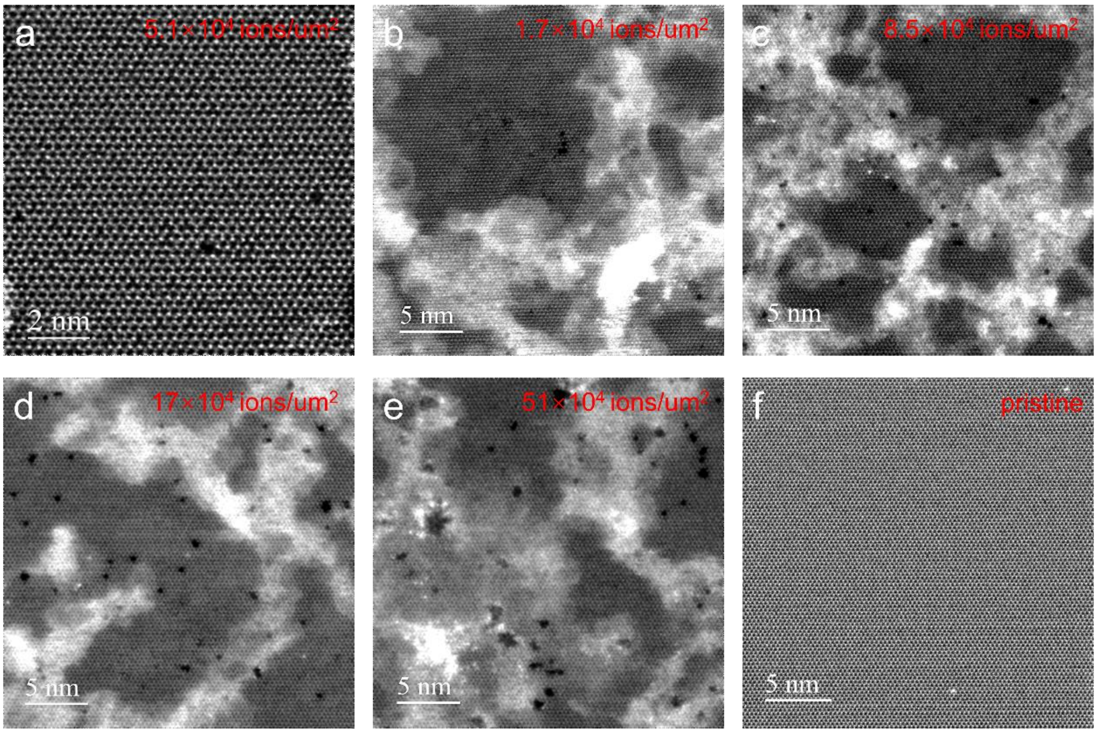

Figure S4. (a) High magnification and (b-e) low magnification HAADF-STEM images of suspended monolayer MoS_2 irradiated by 30 kV Ga^+ with different doses of ion, as well as a low magnification HAADF-STEM image of pristine monolayer MoS_2.

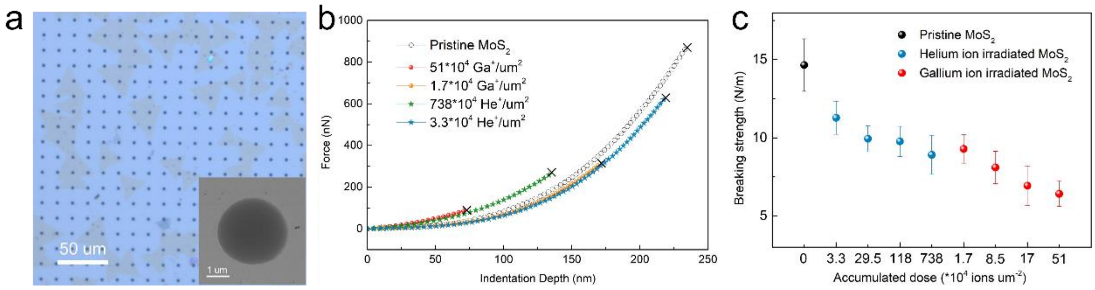

Figure S5. (a) Optical microscope image of MoS_2 on the perforated Si_3N_4 TEM window transferred by PMMA method, and the inset shows a representative AFM image of the suspended monolayer MoS_2. (b) Representative force curve and (c) breaking strength of suspended MoS_2 treated with different doses of He^+ and Ga^+. The symbol × in (b) marks the fracture point.

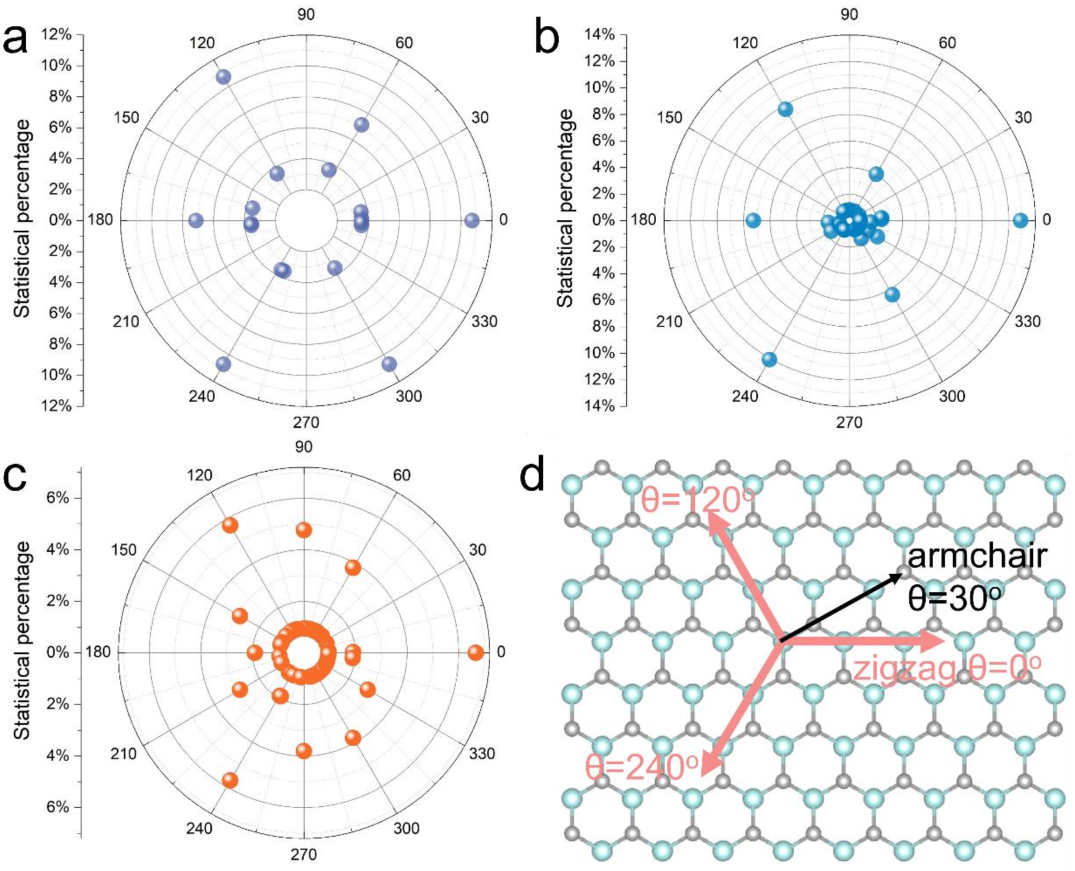

Figure S6. Statistical crack propagation direction in polar coordinates. Angle dependency of the direction of crack propagation in (a) pristine monolayer MoS_2, (b) S vacancy dominated monolayer MoS_2 and (c) MoS_n vacancy dominated monolayer MoS_2. (d) Schematic illustration of propagation angle θ and zigzag direction and armchair direction. It is worth noting that $\theta = 0^o$, 120°, and 240° have the same atomic structure and physical essence due to the triple rotational symmetry of monolayer MoS_2.

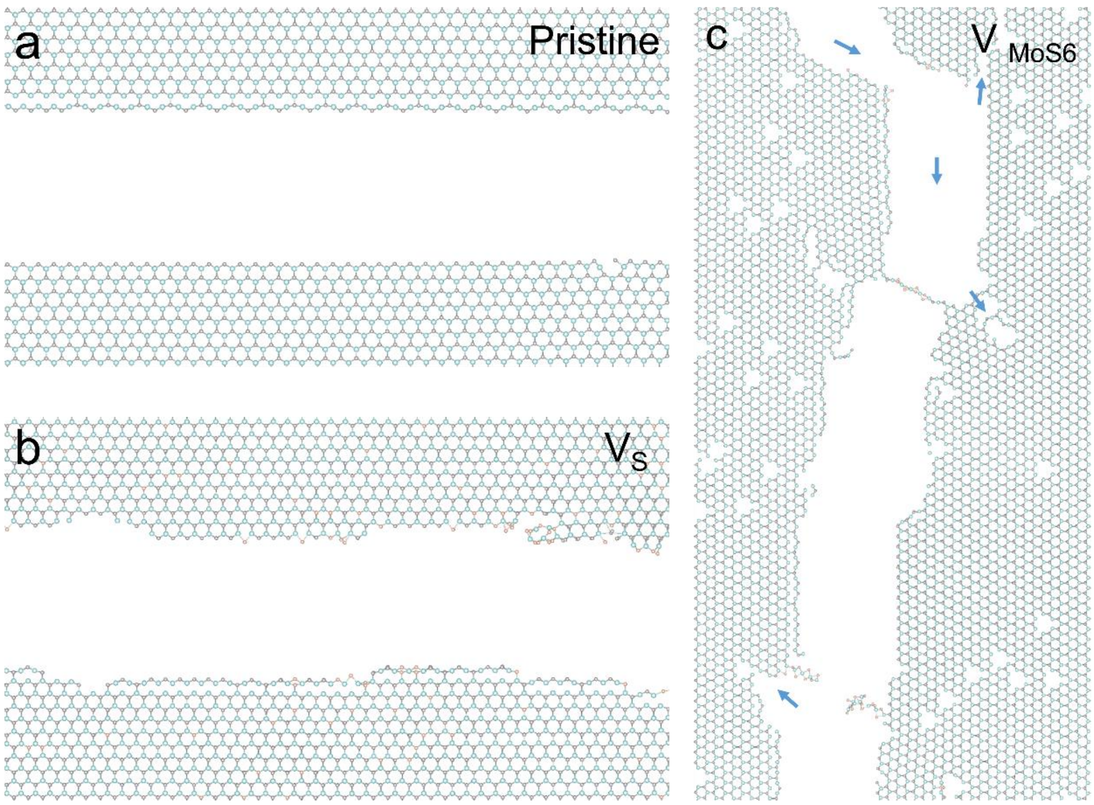

Figure S7. The representative MD simulated atomic structure of (a) pristine MoS_2 after cleavage fracture shows atomically smooth edges along a zigzag direction, (b) fractured monolayer MoS_2 with 3% S vacancy, shows rough and step-like edges, and (c) fractured monolayer MoS_2 with 2% MoS_6 vacancy shows the high-frequency bifurcation of crack path, as highlighted by the blue arrows.

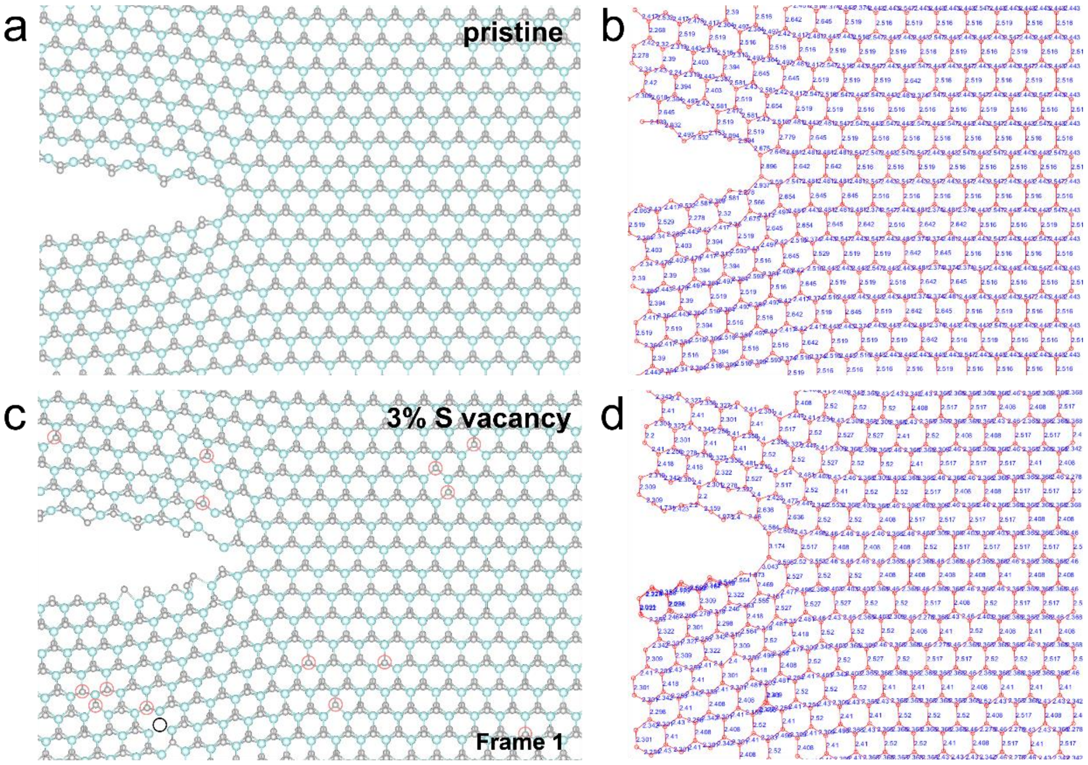

Figure S8. Larger area snapshots of MD simulated crack propagation in (a) pristine MoS_2 and (c) monolayer MoS_2 with 3% S vacancy, and (b, d) their corresponding atomic bond lengths. The black and red circles mark S_2 and S vacancies, respectively.

Table S1. Summary of different defect densities and their corresponding average crack lengths and mechanical characteristics

	pristine	He^+	He^+	He^+	He^+	Ga^+	Ga^+	Ga^+	Ga^+
Ion dose ($\times 10^4$ ions μm^{-2})	0	3.3	29.5	118	738	1.7	8.5	17	51
Density of V_S (1 nm^{-2})	0.12	0.28	0.87	1.29	2.06	0.17	0.31	0.33	holes
Density of V_{MoSn} (1 nm^{-2})	0	0	0	0.04	0.09	0.01	0.05	0.08	holes
Average crack length (μm)	1.1	1	0.8	0.6	0.5	0.5	0.4	0.2	0.1
λ^z_{max} (m N^{-1})	0.29	0.39	0.45	0.46	0.5	0.49	0.55	0.56	0.76

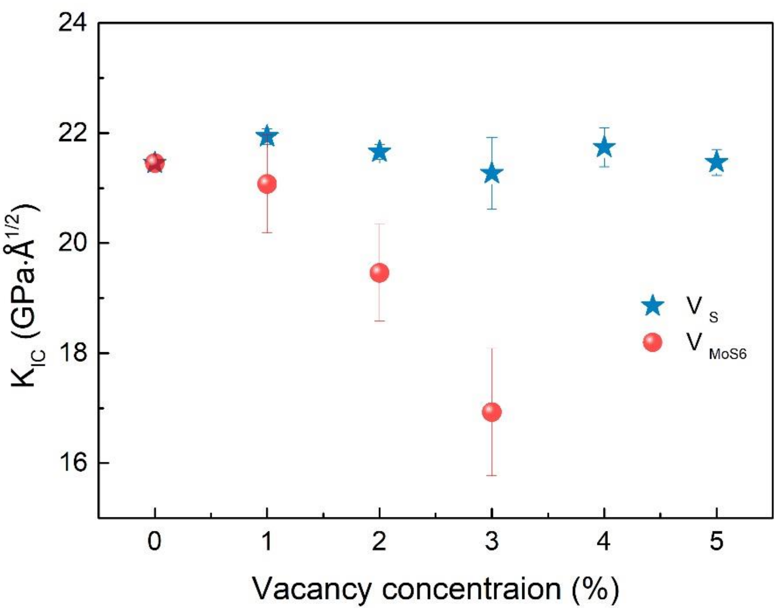

Figure S9. The calculated critical stress intensity factor (K_{IC}) as a function of S and MoS_6 vacancy concentration.
